# Potential Health Benefit of Garlic Based on Human Intervention Studies: A Brief Overview

**DOI:** 10.3390/antiox9070619

**Published:** 2020-07-15

**Authors:** Johura Ansary, Tamara Yuliett Forbes-Hernández, Emilio Gil, Danila Cianciosi, Jiaojiao Zhang, Maria Elexpuru-Zabaleta, Jesus Simal-Gandara, Francesca Giampieri, Maurizio Battino

**Affiliations:** 1 Dipartimento di Scienze Cliniche Specialistiche e Odontostomtologiche—Università Politecnica delle Marche, Via Ranieri 65, 60130 Ancona, Italy; j.ansary@pm.univpm.it (J.A.); d.cianciosi@pm.univpm.it (D.C.); j.zhang@pm.univpm.it (J.Z.); 2 Nutrition and Food Science Group, department of Analytical and Food Chemistry, CITACA, CACTI, University of Vigo-Vigo Campus, 36310 Vigo, Spain; tforbes@uvigo.es; 3 Nutrition and Food Science Group, Department of Biochemistry, Genetics and Immunology, Faculty of Biology, University of Vigo, 36310 Vigo, Spain; egil@uvigo.es; 4 Dipartimento di Scienze Cliniche e Molecolari, Facoltà di Medicina, Università Politecnica delle Marche, 60131 Ancona, Italy; p015008@staff.univpm.it; 5 Nutrition and Bromatology Group, Department of Analytical and Food Chemistry, Faculty of Science, University of Vigo, Ourense Campus, E-32004 Ourense, Spain; jsimal@uvigo.es; 6 College of Food Science and Technology, Northwest University, Xi’an, Shaanxi 710069, China; 7 International Research Center for Food Nutrition and Safety, Jiangsu University, Zhenjiang 212013, China

**Keywords:** garlic, sulfur-containing compounds, polyphenols health benefits, metabolism and bioavailability

## Abstract

Garlic is a polyphenolic and organosulfur enriched nutraceutical spice consumed since ancient times. Garlic and its secondary metabolites have shown excellent health-promoting and disease-preventing effects on many human common diseases, such as cancer, cardiovascular and metabolic disorders, blood pressure, and diabetes, through its antioxidant, anti-inflammatory, and lipid-lowering properties, as demonstrated in several in vitro, in vivo, and clinical studies. The present review aims to provide a comprehensive overview on the consumption of garlic, garlic preparation, garlic extract, and garlic extract-derived bioactive constituents on oxidative stress, inflammation, cancer, cardiovascular and metabolic disorders, skin, bone, and other common diseases. Among the 83 human interventional trials considered, the consumption of garlic has been reported to modulate multiple biomarkers of different diseases; in addition, its combination with drugs or other food matrices has been shown to be safe and to prolong their therapeutic effects. The rapid metabolism and poor bioavailability that have limited the therapeutic use of garlic in the last years are also discussed.

## 1. Introduction

Spices are not only used to increase the aroma, flavor, and color of food, but are also considered for therapeutic purposes for their potential prevention of different acute and chronic diseases. Various bioactive compounds of spices, including alkaloids, tannins, vitamins and phenolic diterpenes, flavonoids and polyphenols, as well as sulfur-containing compounds, are responsible for different types of therapeutic properties, thanks to their antioxidant, anticarcinogenic, antitumorigenic, anti-inflammatory, and glucose and cholesterol-lowering properties [1].

Garlic (*Allium sativum* L.), a member of the Amaryllidaceae family that is cultivated all over the world, provides noteworthy health benefits. In 1550 B.C., antibiotics and pharmacy products were not available so garlic was used for medicinal purposes in different epidemics, such as typhus, dysentery, cholera, and influenza [2]. The therapeutic effects of garlic are mainly due to the impressive activity of its bioactive compounds, such as organic sulfides [3], saponins [4], phenolic compounds [5], and polysaccharides [6]. For example, several in vitro and in vivo studies have showed that garlic compounds are able to modulate various signaling pathways, including nuclear factor-κB and wingless-related integration site [7], matrix metalloproteinases, nuclear factor erythroid 2 like 2, protein kinase B (pAkt), mitogen-activated protein kinase, c-Jun N-terminal kinases, caspases, p38, transforming growth factor beta 1 (TGF-β1), TGF-β type II receptor, psmad2/3, smad4 and smad7 [8,9], cytokines, intercellular adhesion molecule [10], notch pathway [11], 5’ AMP-activated protein kinase pathway [12], vascular endothelial growth factor [13], cyclooxygenase 2, inducible nitric oxide synthase, Akt/mTOR [14], and Keap1 [15], leading to improved anti-inflammatory and antioxidant properties, as well as chemopreventive, antiproliferative, anti-angiogenic, antidiabetic, and cardioprotective effects. However, it should be taken into account that these compounds have a very low availability. For example, in a randomized controlled trial, after oral administration of 1 g or 3 g of dehydrated garlic powder, the organosulfur compound diallyl disulfide (DADS) and diallyl sulfide (DAS) were not detected in the urine samples after 6 and 24 h [16]. Furthermore, allyl thiosulfates of garlic preparation undergo extensive metabolism, producing allyl methyl sulfide, essentially present in breath [17]. 

Thus, the present review aims to provide a comprehensive overview of clinical trials in the last 20 years, assessing the therapeutic effects of garlic on different common human diseases, such as cancer, cardiovascular pathologies, diabetes, metabolic disorders, osteoporosis, and skin diseases, mostly focusing on its antioxidant, anti-inflammatory, and lipid lowering effects. This review also presents the main molecular mechanisms of garlic involved in its promising health benefits after consumption. 

## 2. Literature Search

A comprehensive revision was done, using electronic databases, including Medline, Scopus, Google Scholar, and Web of Science, and related clinical trials regarding garlic effects on human diseases have been considered. The keywords “garlic”, “garlic effects”, “garlic bioactive compounds”, “garlic polyphenols”, “allicin”, “alliin”, “allylpropyl disulfide”, “diallyl trisulfide (DATS)”, “ajoene”, “S-allylcysteine”, “S-allylmercaptocysteine” (SAMC), “vinyldithiins”, “aged garlic”, “garlic cloves”, “garlic metabolism”, “garlic absorption”, “garlic bioavailability”, “garlic antioxidant capacity”, “garlic anti-inflammatory property”, “garlic lipid lowering effect”, as well as garlic with each specific disease (i.e., “garlic and cancer”, “garlic and colon cancer”, “garlic and prostate cancer”, “garlic and esophageal cancer”, “garlic and larynx cancer”, etc.) have been used. All the articles included have been published from 2000–2020.

## 3. Bioactive Compounds of Garlic

Garlic is considered as a functional spice because of its diverse array of nutritional constituents, phytochemicals, and fiber. It contains high levels of potassium, phosphorus zinc, and sulfur, moderate levels of selenium, calcium, magnesium, manganese, iron, and low levels of sodium, vitamin A and C and B-complex [18]. In the last years, considerable attention has been given to its main bioactive compounds, particularly polyphenols, flavonoids, flavanols, tannins [5,19], saponins [4], polysaccharides [6], sulfur-containing compounds (including alliin, allicin, ajoene, allylpropyl disulfide, DATS, S-allylcysteine, vinyldithiins, SAMC), enzymes (like allinase, peroxidase, myrosinase), and other compounds, such as β-phellandrene, phellandrene, citral, linalool, and geraniol [3]. There are more than twenty well-known polyphenolic compounds in garlic, including kaempferol 3,7-di-*O*-rhamnoside, kaempferol-3 glucuronide, kaempferol-3-*O*-glucoside, kaempferol-3-*O*-beta-d-glucoside-7-*O*-alpha-l-rhamnoside, luteoline, and apigenine [20]. Moreover, it contains 17 amino acids with eight main amino acids [21]. Generally, bioactive compounds are present in intact garlic, but, after chopping or crushing, a higher number of compounds, such as allicin, DAS, DADS, dithiins, and ajoene have been found after different types of chemical reactions [22,23].

## 4. Metabolism and Bioavailability

**Figure 1:**
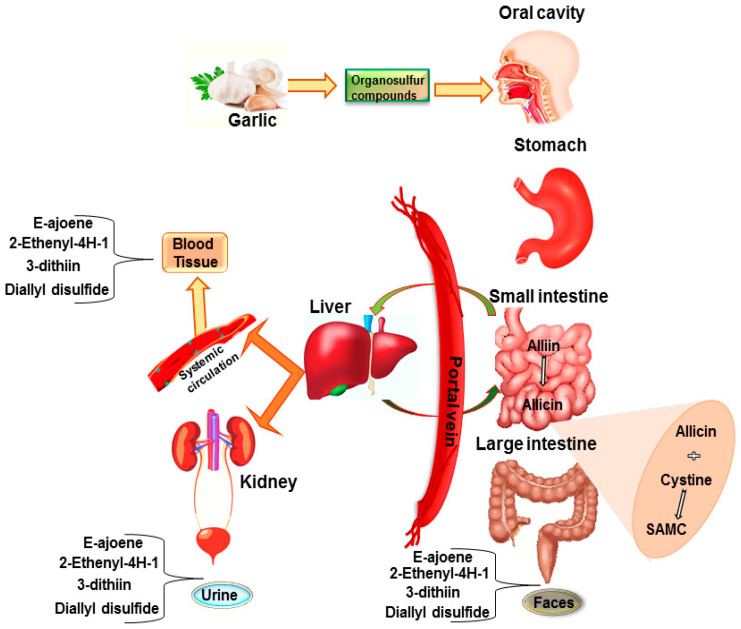
Schematic illustration of absorption, metabolism, and distribution of garlic organosulfur compounds in the gastrointestinal (GI) tract. The absorption of garlic occurs in the GI tract where allicin is released from alliin and contacts with cystine that is released from protein diet, forming S-allylmercaptocysteine (SAMC). After metabolism, the secondary metabolites of allicin including E-ajoene, 2-ethenyl-4H-1, 3-dithiin, and diallyl disulfide (DADS) are available in blood, urine, and faces.

Based on several in vitro studies for cancer treatment, a large number of in vivo and clinical studies have been conducted on raw garlic and/or its formulation, although results are conflicting. Indeed, the main sulfur-containing groups exhibit different bioavailability between raw garlic and specific garlic supplement formulations. For example, the bioavailability of allicin from nine garlic-based food and 13 garlic supplements was tested on 13 subjects measuring the concentration curve of breath allyl methyl sulfide, the most important garlic metabolite, highlighting a higher bioavailability of allicin from garlic supplements than that of crushed raw garlic [24]. In crushed raw garlic cloves, allicin is liable for most of the pharmacological activity and it is metabolized immediately under enzyme-inhibiting gastrointestinal conditions (half-life <1 min) to allyl-mercaptan. After consuming a large amount (25 g) of crushed raw garlic, allicin and its metabolites are available in the blood, urine, and stool [25] (Figure 1). Similarly, after intravenous injection, allicin rapidly disappears from circulation and is transformed into secondary metabolites, including E-ajoene, 2-ethenyl-4H-1, 3-dithiin, and DADS [26]. On the other hand, allicin bioavailability of enteric tablets varies from 36% to 104% at ≥0.5 h after garlic product consumption, which was decreased from 22% to 57% in breath, when eating with a high protein meal. Independent of meal type, garlic capsules gave 26–109% lower bioavailability, while non-enteric tablets showed 80–111% higher bioavailability [24]. 

Additionally, protein derivative cystine interacts with allicin quantitatively at body temperature to form two equivalents of SAMC. This probably happens when cysteine is released from digested meal protein and comes in contact with allicin released from garlic products in the gastrointestinal tract. In addition to this, after an oral administration of 200 mg/kg of DADS in rats, the main metabolites, such as allyl methyl sulfoxide and allyl methyl sulfone, were found in plasma, stomach, liver, and urine [27].

Aged garlic extract (AGE) contains primarily water-soluble organosulfur compounds, for example, S-allyl cysteine (SAC) and SAMC that have different pharmacokinetic behaviors than oil-soluble organosulfur compounds [28]. After garlic oral administration, SAC is absorbed immediately from the gastrointestinal tract (GI) tract, its half-life is more than 10 h and the renal clearance is more than 30 h in humans. The result after the evaluation of the safety and efficacy of SAC illustrated that it seems to play a key role in the biological effects of garlic [29].

Finally, extraction may improve the bioavailability of the whole garlic as well as of different crude ingredients and reduce toxicities. For example, in AGE, during the extraction process, the odorous, harsh, and irritating compounds of garlic are naturally transformed into stable and safe sulfur compounds; moreover, different toxicological studies have confirmed the safety of aged garlic [30]. 

## 5. Clinical Trials on Garlic

Recently, garlic consumption has gained particular attention due to its therapeutics properties against cancer, cardiac disease, blood pressure, diabetes, bone and skin diseases, and other pathologies, thanks to its antioxidant, anti-inflammatory, and lipid-lowering effects. All clinical studies are summarized in Table 1, Table 2, Table 3 and Table 4

### 5.1. Garlic Properties 

#### 5.1.1. Antioxidant Capacity

Garlic has strong antioxidant properties due to its nutritional and phenolic compounds [31]. For example, the antioxidant properties of aged garlic extract (AGE) decrease reactive oxygen species, which are produced through increased metabolism or chronic inflammation, thus preventing the endothelial dysfunction, an early marker of atherosclerosis [32]. In a randomized double-blind placebo-controlled nutritional intervention, garlic extract (GE) intake at 400 mg/day for three months enhanced antioxidant status, reducing in turn the cardiovascular risk in obese patients through modulating endothelial biomarkers, such as C-reactive protein (hs-CRP), low-density lipoprotein cholesterol (LDL), high-density lipoprotein (HDL) levels, triglycerides (TGs), and plasminogen activator inhibitor-1 (PAI-1) [33]. Moreover, in diabetic patients after 30 days of supplementation with 3.6 g garlic clove per day, enhanced antioxidant activities, such as superoxide dismutase (SOD), catalase (CAT), and glutathione peroxidase (GPx) activities, significantly increased in circulating human erythrocytes compared with control [34]. Recent meta-analysis of clinical trials demonstrated that garlic supplementation modulates oxidative stress markers, including total antioxidant capacity (TAC) and malondialdehyde (MDA) [35]; for example, the garlic tablet (Garlet) exerted a significant role on oxidative stress via decreasing MDA levels and improving TAC concentration in postmenopausal osteoporotic women at a dose of 1200 μg allicin daily for one month compared with placebo [36]. Furthermore, garlic administration synergistically improved (resistance and endurance) the training effect against oxidative stress by modulating oxidative stress markers, such as TAC and MDA, after eight weeks of treatment at a dose of 250 mg garlic capsule per day for eight weeks [37]. However, another double-blind crossover pilot study conducted on type 2 diabetic patients revealed that AGE has no significant effect at 1200 mg per day on the oxidative stress in endothelial tissue after four weeks treatment [38]. Similar results were found in a randomized crossover study, where in men with coronary artery disease, the administration of AGE supplementation at 2.4 g per day for two weeks did not change markers of oxidant stress and systemic inflammation significantly during the study, even if an improvement of the flow mediated endothelium-dependent dilation from baseline and of endothelial function were found [39].

Finally, a few crossover studies showed that the consumption of a high antioxidant spices diet, including garlic, improved antioxidant status in cancer patients and postprandial lipemia in healthy overweight adults compared to low spices meal [40,41]. In addition, consumption of 400 mg garlic and 1 mg allicin per day decreased oxidative stress after nine weeks in pregnant women at risk for pre-eclampsia but had no significant effect on TAC and pregnancy issues [42]. 

#### 5.1.2. Anti-Inflammatory Properties 

Various chronic diseases, such as cancer and cardiovascular diseases, are related with inflammatory processes; in these conditions, different types of therapeutic and natural tools have been used to prevent them [43]. In this context, garlic has shown to exert potent anti-inflammatory effects by decreasing the inflammatory biomarkers in end-stage renal disease and adult patients. A double-blind randomized clinical trial showed a significant reduction of inflammatory cytokines, such as interleukin 6 (IL-6), C-reactive protein (CRP), and erythrocyte sedimentation rate when standardized GE was administered at 400 mg twice a day for eight weeks in peritoneal dialysis patients [44,45]. In addition, a meta-analysis revealed that garlic supplementation, including AGE, garlic powder and garlic capsule, reduced serum concentrations of tumor necrosis factor alpha (TNF-α), and CRP, but did not affect serum adiponectin and leptin in healthy adults [46]. 

Immune cells are responsible for the anti-inflammation effect; aged garlic contains various compounds that can improve immune systems by modulating cytokine production. For example, the consumption of aged garlic supplementation at a dose of 2.56 g per day for 90 days increased the activity of immune cells, such as γδ-T and natural killer (NK) cells and decreased inflammation by reducing TNF-α and IL-6 in obese adults [47]. Interestingly, the same dose of GE boosted immune cell function, decreasing the severity of cough and flu [48] and increasing urinary cytokine IL-12 excretion, even if no significant effect on IL-8 and TNF-α were found [16]. In addition, a negative correlation has been found between organosulfur compounds of AGE and obesity-induced inflammation in a randomized, double-blind, placebo-controlled clinical trial. After taking AGE supplement at a dose of 3.6 g per day for six weeks, SAC reduced obesity-induced inflammation by releasing hydrogen sulfide (H_2_S) via increasing its endogenous products [49]. Moreover, garlic supplementation increased microbial richness and diversity and improved inflammation condition, in patients with uncontrolled hypertension [50], while no significant effects have been found after garlic consumption at 2.1 g per day for 12 weeks, on inflammation in overweight subjects [51] and type 2 diabetes patients with high cardiovascular risk [38].

#### 5.1.3. Lipid Lowering Effect

Garlic has shown promising lipid-lowering effects on hyperlipidemic patients through the reduction of serum cholesterol concentration [52]. In diabetic patients the combination of garlic with olive oil effectively regulated serum cholesterol and triglycerides levels, as well as dyslipidemia [53]. Silagy and Neil (1994) suggested that garlic in non-powder and powder forms undoubtedly reduced serum lipids levels over a one to three month period. After four months, the consumption of GE raised HDL and lowered LDL and cholesterol levels in 23 hyperlipidemic patients [54]. Several studies showed that the administration of aged black garlic or garlic tablet at a dose 300 mg or 6 g two times per day for 4 or 12 weeks reduced the levels of total cholesterol (TC), triglycerides, and LDL while it elevated that of HDL in patients with mild hypercholesterolemia and dyslipidemia or type 2 diabetics [55,56,57]. Moreover, aged garlic reduced TC and TG at a dose of 2.4 g per day for two weeks in patients with coronary artery disease [39]. In another randomized, double-blind, placebo-controlled trial, 10.8 mg per day of garlic powder tablet for 12 weeks reduced the triacylglycerol concentration in healthy volunteers [58]. Furthermore, enteric-coated garlic powder tablet containing 400 mg garlic and 1 mg allicin two times daily reduced cholesterol and LDL levels, showing protective effects in a single-blind study with 150 hypercholesterolemia patients [59].

### 5.2. Garlic Therapeutic Effects against the Main Common Human Disease

#### 5.2.1. Cancer

Cancer is one of the main causes of deaths worldwide. Based on National Cancer Database and the Surveillance, Epidemiology, and End Results, about 16.9 million people were identified with cancer in 2019 and this number will probably rise to more than 22.1 million in 2030 [60]. The Food and Drug Administration’s evidence-based review system for the scientific evaluation of health showed no reliable evidence for the relation between garlic and a reduced risk of gastric, breast, and lung cancer [61]. However, credible evidence for an association between garlic intake and colon, prostate, esophageal, larynx, oral, ovary, and renal cell cancers has been reported, even if all studies were observational and the number of such trials that are scientifically considered valid in this analysis is remarkably few and the number of subjects involved generally small. As a result, relations between garlic and reduction of risk of cancers are still uncertain [62,63]. Interestingly, garlic can provide symptomatic relief of various cancer conditions, including breast, colorectal, colon, gastric, lung, and pancreatic cancers. It may be a therapeutic potential for specific cancer treatment that has been reported in human-based clinical studies, presented and discussed in Table 2. In this context, personalized diets with supplemented functional elements, including functional phytochemicals, such as allyl sulfur compounds or allicin, have provided high amounts of antioxidants to patients in chemotherapy and in remission [40]. A randomized controlled trial showed that dietary intervention for six months among breast cancer survivors increased adherence to a Mediterranean style diet and consequently raised the consumption of anti-inflammatory spices, such as garlic [63]. Another randomized double-blind factorial trial on garlic highlighted a decreased appearance of precancerous gastric lesions or gastric cancer [64], while consumption of 200 mg of synthetic allitridum (diallyl trisulfide) with 100 mg of selenium reduced gastric cancer risk [65]; similar results were found with 7.3 years consuming garlic supplementation at a dose of 200 mg caps or steam-distilled garlic oil 1 mg two times per day that reduced advanced gastric lesions [64]. In addition, long-term consumption of garlic, garlic supplements or garlic with vitamins reduced gastric cancer [66], precancerous gastric lesions [67], and mortality rate [68]. Garlic supplementation at a dose of two capsules two times a day for 7.3 years increased serum folate and improved moderate folate deficiency in patients with gastric lesions in rural Chinese populations [69]. In addition, the intake of garlic supplements <0.60 to >3.65 kg per year for two years was significantly associated with decreased risk of colorectal adenoma, which is a precursor of colorectal cancer (CRC) [70,71]. Epidemiological studies of randomized controlled trials explained that the administration of GE decreased colon adenomas and CRC in patients with CRC [72] via increased NK cell activity [73]. Correspondingly, AGE at a dose of four caps per day for six months prevented the reduction of NK cell number in patients with liver and pancreatic cancer [73]. 

Few epidemiological studies conducted in the Chinese population found a significant inverse relation between consumption of raw garlic or garlic components 8.4 g or 33.4 g per week for seven years and lung cancer [74]. Finally, GE showed preventive effect on febrile neuropathy after receiving chemotherapy in patients with hematological malignancies, potentially reducing the risk of chemotherapy related febrile neutropenia after receiving GE at 900 mg per day for three weeks compared with placebo [75].

Numerous mechanisms have been recommended to explain the chemo-preventive effects of garlic, including the inhibition of DNA adduct formation, the inhibition of mutagenesis by blocking metabolism, through its free-radical scavenging, or by decreasing cell proliferation and tumor growth [74]. In this context, Charron et al. [76] performed a clinical trial on gene expression related to immunity, apoptosis, and xenobiotic metabolism in humans, after consumption of 5 g raw, crushed garlic daily for 10 days. A single meal containing raw crushed garlic activated the expression of seven genes, such as activating protein with immunoreceptor tyrosine-based activation motif 1, aryl hydrocarbon receptor nuclear translocator, aryl hydrocarbon receptor, hypoxia-inducible factor 1α, c-Jun, nuclear factor of activated T cells, oncostatin M and V-rel avian reticuloendotheliosis viral oncogene homolog in blood of healthy volunteers, thus inhibiting tumorigenesis. 

#### 5.2.2. Cardiovascular Diseases

Currently, cardiovascular disease (CVD) represents the major cause of morbidity and death worldwide, with 17.3 million deaths per year, a number that is anticipated to rise to over 23.6 million by 2030 [77]. Many risk factors affect the development of CVD, including type 2 diabetes mellitus, obesity, resistance to insulin, high blood pressure, metabolic syndrome and high serum triglycerides levels and plasma lipid profile [78]. Based on current research, garlic can significantly reduce the risk of atherosclerosis, hypertension, diabetes, hyperlipidemia, myocardial infarction, and ischemic stroke [79], thanks to the synergistic effects of its nutritional and phytochemical components. For example, atherosclerosis and vascular inflammation are usually accompanied with oxidative stress, endothelial dysfunction, and inflammatory cytokines [80,81]. From a dietary approach, garlic has the potential role in the prevention and treatment of atherosclerosis and myocardial infarction [82,83], as demonstrated by a randomized trial performed with AGE on adipose tissue surrogates for coronary atherosclerosis progression, that reported a decrease of the coronary atherosclerosis growth at dose of 250 mg AGE daily for 12 months by reducing epicardial adipose tissue, pericardial adipose tissue, periaortic adipose tissue, and subcutaneous adipose tissue [84]. In addition, AGE prevented atherosclerosis process by developing microcirculation in patients at a dose of 2400 mg AGE per day [85]. Moreover, isoflavonoid-rich garlic herbal preparation blocked atherosclerosis progression 1.5-fold in postmenopausal women at a dose of 500 mg for 12 months after administration [86]. Another randomized double-blind study demonstrated that 2400 mg AGE per day for 52 weeks decreased low attenuation plaque in coronary arteries of patients with metabolic syndrome [87], but no significant effect on lipoprotein levels was highlighted [88]. On the other hand, AGE at 6 g daily for 12 weeks reduced the levels of lipoprotein B and raised HDL levels, showing cardioprotective effect in patients with mild hypercholesterolemia [55]. In addition, GE administrated at 400 mg per day for three months modified the markers of endothelial function such as hs-CRP, PAI-1, cholesterol (total, LDL, HDL) and triglycerides as well as suppressed chronic inflammation in obese individuals [33], probably also modulating by ATP-binding cassette (ABC) A1 or ABCG1 expressions in peripheral blood mononuclear cells (PBMCs) [89]. Another meta-analysis suggested that garlic has a cardioprotective effect by decreasing serum TC and TG levels in patients with mild hypercholesterolemia [90].

Finally, even if several experimental studies demonstrated that garlic exerts antiplatelet properties, a randomized clinical trial suggested that garlic oil and tablet have little or no effect on the aggregation of platelets and showed mild adverse effects by increasing bleeding in some tested volunteers, even if the tablet dose might be equivalent to the dose of cardio-protective agent aspirin [91,92]. Thus, substantial attention is being given to the assurance of using GE along with oral anticoagulation therapy. A double-blind, randomized, placebo-controlled pilot study showed that 5 mL of AGE two times per day may be safe for patients with hemorrhages when combined with warfarin therapy [93]. 

#### 5.2.3. Metabolic Syndrome

Metabolic syndrome is a cluster of metabolic diseases, including abdominal obesity, hypertension, atherogenic dyslipidemia, prothrombotic, and proinflammatory conditions and the risk of this disease increases approximately five-fold and two-fold in patients affected by type 2 diabetes mellitus and cardiovascular disease, respectively [94,95]. 

In this context, the consumption of raw crushed garlic at 100 mg two times per day for four weeks significantly decreased several risk factors of metabolic syndrome, including blood pressure, triglyceride levels, fasting blood glucose, as well as improved serum high-density lipoprotein cholesterol [95]. Moreover, a double-blinded placebo-controlled study revealed that the treatment with garlic tablet Allicor at a dose of 300 mg two times a day for four weeks along or combined with sulfonylureas drugs led to excellent metabolic control by lowering fasting blood glucose, serum fructosamine, and serum triglyceride levels in patients affected by type 2 diabetes mellitus, also decreasing cardiovascular risk [56]. In addition, administration of garlic clove for 30 days in type 2 diabetic patients reduced blood glucose and lipids metabolism and reduced the serum lipid such as cholesterol, TG, and LDL but improved HDL fraction [34]. Similarly, administration of 100 mg daily garlic for five months and 300 mg garlic twice daily for 24 weeks in diabetic patients decreased blood glucose, cholesterol, and TG and increased HDL levels [96]. Another study revealed that AGE reduced the risk factors of metabolic syndrome at a dose of 1.2 g per day for 24 weeks through increased plasma adiponectin levels in patients, without any side effects [97]. Accordingly, AGE reduced low attenuation plaque in coronary arteries of patients with metabolic syndrome after consumption of 2400 mg AGE per day [87], highlighting its antidiabetic, anti-lipidic, and antioxidant properties [96].

#### 5.2.4. Blood Pressure

When blood force gives pressure against blood vessels or arteries walls hypertension is developed [98]. Recently, garlic showed a satisfactory effect as a hypertensive remedy by regulating high cholesterol levels and stimulating the immune system [99]. A dose-response trial reported that AGE had an antihypertensive effect by lowering systolic blood pressure in the case of uncontrolled hypertension without any remarkable side effects [100]. Moreover, in moderately hypercholesterolemic subjects the administration of AGE extracts or dried garlic powder at 7.2 g per day for four weeks decreased systolic blood pressure (SBP) and diastolic blood pressure (DBP) moderately (5.5%) through decreasing serum TC, and LDL, even if no prominent change in HDL was found compared with placebo [101,102]. A similar result was observed with raw crushed garlic at 100 mg/kg two times per day, that reduced SBP and DBP via downregulation of TG level and upregulation of serum HDL cholesterol after four weeks consumption in patients with metabolic syndrome [96]. Furthermore, administration of garlic homogenate-based supplementary diet at 300 mg per day for 12 weeks significantly reduced SBP and DBP in mild hypertension patients but not in prehypertension patients, without any clinical side effects [103]. 

In addition, the combined intake of garlic and coriander exerted a significant effect on lipid profile, where garlic, coriander or their mixture intake at a dose of 2 g/day highly modulated body mass index, TC, LDL, and HDL and decreased blood pressure in hypertensive patients [104]. In contrast, Simons et al. found no significant outcomes of garlic supplementation on lipid and lipoprotein levels in patients with mild hypercholesterolemia [105].

#### 5.2.5. Diabetes

Diabetes is one of the major non-communicable life threatening chronic and pervasive conditions. It results from an absolute or relative deficiency or resistance to insulin [79], remaining a prominent chronic disease with the number of diabetics quadrupling in the last three decades in the world [106,107]. The International Diabetes Federation estimated that 415 million adults had diabetes in 2015, and it is projected to reach 642 million within 2040 [108]. Oxidative stress is responsible for promoting diabetes and preclinical studies showed that garlic active organosulfur compounds reduced hyperglycemia via improving the antioxidant status in circulation of diabetic rats [106]. In addition, the garlic component acts as hydrogen sulfide donors which also control type 2 diabetes [109]. Recently, few meta-analyses demonstrated that garlic may decrease lipid profile and glucose parameters such as fasting blood glucose concentrations [110,111] and hemoglobin A1c (HbA1C) in diabetics patients [112]. In addition, uptake of 300 mg garlic supplementation two times per day for 12 weeks significantly improved serum TG, TC, and LDL level and decreased the serum lipid level compared with placebo diabetic patients with uncontrolled dyslipidemia [113]. Moreover, the combination of antidiabetic drug metformin at 500 mg two times per day with garlic at 300 mg three times per day for 24 weeks had more potential in the management of patients with diabetes by reducing total cholesterol, LDL, and TG and improving hyperlipidemia [114]. On the other hand, a double-blind, placebo-controlled crossover pilot study showed that AGE had no significant effect on insulin resistance at 1200 mg per day for four weeks in adults with type 2 diabetes [37]. Finally, a double-blind clinical trial in diabetic patients revealed that herbal medicine containing garlic at a dose of 750 mg 3 times per day for 12 weeks had potential effects for diabetes management by reducing fasting glucose blood levels through the decrease of HbA1 [115]. 

#### 5.2.6. Bone Disease

Osteoarthritis (OA) is an extensive degenerative disease of bone joints, which is related to chronic and disabling pain, where adipocytokines, resistin, and proinflammatory markers have a particular role in its pathogenesis [116]. Garlic supplement at 1000 mg per day has been shown to be effective on symptom relief in overweight or obese women with knee osteoarthritis, after 12 weeks of administration [117]. Moreover, the intake of garlic tablet at 500 mg two times daily for 12 weeks showed anti-inflammatory and analgesic effects by reducing serum resistin and TNF-α concentrations and pain severity in obese or overweight women with knee OA [116].

Another randomized clinical trial revealed that garlic tablet acts as an antioxidant in postmenopausal osteoporotic women. In this study, a significant decrease in advanced oxidation protein products and plasma protein carbonyl plasma levels and a concomitant increase in TAC as well as a reduction of oxidative stress and osteoporosis were found after garlic tablet consumption at a dose of 2 tablets (equivalent 2 g fresh garlic) per day for 12 months [118]. In addition, a correlation was found between pro-inflammatory cytokine activity and garlic tablet administration two times daily for eight months in postmenopausal bone loss, where garlic modulated cytokine production and reduced osteoporosis in postmenopausal osteoporotic women [36].

#### 5.2.7. Skin Disease

Garlic has long been used in traditional and complementary medicine [119] and several clinical trials have demonstrated the efficacy of its administration or application in resolving symptoms associated with warts [120], denture stomatitis [121], venous ulcers [122], and skin wounds [85,123]. For example, in preclinical studies AGE showed wound healing potential in a dose-dependent manner after six days application [123,124].

Treatment of wart virus by improving immune system might be accomplished by intralesional immunotherapy [125,126] and it may damage all sores on the body [127]. A randomized control study showed that lipid portion of GE two times daily for four weeks had greater potential on patients with recalcitrant multiple common warts compared to any other treatment, through the modulation of TNF-α serum level as well as the promotion of immunotherapy [120]. Similarly, denture stomatitis expresses a common form of chronic oral candidiasis and a randomized clinical trial study demonstrated that GE at 40 mg/mL three times per day could be a potential substitution for denture stomatitis treatment compared with nystatin [121]. Finally, a prospective non-randomized pilot study was performed on venous ulcer patients with herbal ointment containing garlic, showing anti-erythematous, epithelizing, and anti-edematous properties and decreased the venous ulcer area after seven weeks of application [122].

#### 5.2.8. Other Diseases

In recent years, antibiotic-resistance against microorganisms has become a serious issue, thus substantial attention is being given to the antimicrobial activities of spices, such as garlic, because of their inhibitory effects against pathogenic viruses [128], bacteria [129], yeast [130], and fungi [131]. The antimicrobial mechanism of garlic may include the inhibition of extracellular enzymes, the deprival of the substrates needed for microbial growth, the morphological change and the anti-adherence of bacteria to epithelial cells [132]. For example, a randomized double-blind controlled clinical trial found an inversed relation between *Streptococcus mutans*, *Lactobacilli* species, and *Candida albicans* and garlic with lime containing mouth rinses in children with severe early childhood caries [133]. In addition, garlic tablet Garcin at a dose of 1500 mg per day for seven days has been shown to be effective for the treatment of Candida vaginitis instead of fluconazole in women with vaginitis [134]. In contrast, another randomized placebo controlled double-blind trial showed adverse effects of garlic in asymptomatic women with culture-positive on vaginal candida colony counts at dose of 350 mg garlic tablets two times per day [135]. Moreover, in the case of acute respiratory viral infection, garlic tablet Allicor at 600 mg per day for five months diminished acute respiratory diseases morbidity 2–4-fold at the first stage and 1.7-fold at the second stage compared to the controls, as a consequent inhibition of infection [136]. 

Garlic tablets at dose 400 mg daily are also a promising candidate for nosocomial infections in hospitalized patients in intensive care units and might be used for the prevention of septicemia and urinary tract infections after six days of treatment [137]. Furthermore, laboratory studies and clinical trials demonstrated that GE contains organosulfur compounds that exhibit antileishmanial and immunomodulatory activity [138]. For example, garlic topical gel showed anti-leishmanial activity in cutaneous leishmaniasis patients and recovered the lesions after applying topical gel for six and eight weeks [139]. In contrast, another pilot randomized controlled trial on garlic capsule showed no significant effect with minor adverse effects against Pseudomonas aeruginosa quorum sensing in cystic fibrosis patients after daily treatment for eight weeks [140]. 

Garlic intake exerts a protective effect also in gastric diseases [141]. Panjeshahin et al. did a metanalysis on human liver enzyme and evidenced that garlic supplementation significantly reduced liver enzyme aspartate transaminase levels without showing any effect on alanine aminotransferase (ALT) level [142]. In addition, 1.5 g of fermented GE administration per day effectively and safely improved hepatic function by ameliorating serum gamma-glutamyl transpeptidase (GGT) and ALT levels in adult patients over a 12-week period [141]. Similarly, garlic formulation of dimethyl-4, 4′-dimethoxy-5, 6, 5′, 6′-dimethylene dioxybiphenyl-2, 2′-dicarboxylate (DDB) plus garlic oil (GO) gave a significant hepatoprotective effect in patients with chronic hepatitis, after oral administration. Futhermore, six weeks of treatment of three to six capsules per day of DDB plus GO reduced the action of ALT in serum and improved the condition of chronic hepatitis [143]. Finally, a population-based study found that frequent consumption of raw garlic is inversely connected with nonalcoholic fatty liver disease (NAFLD). In patients with NAFLD, garlic powder consumption modulated body components by reducing body weight and fat mass [144,145].

## 6. Conclusions and Future Perspectives

**Figure 2:**
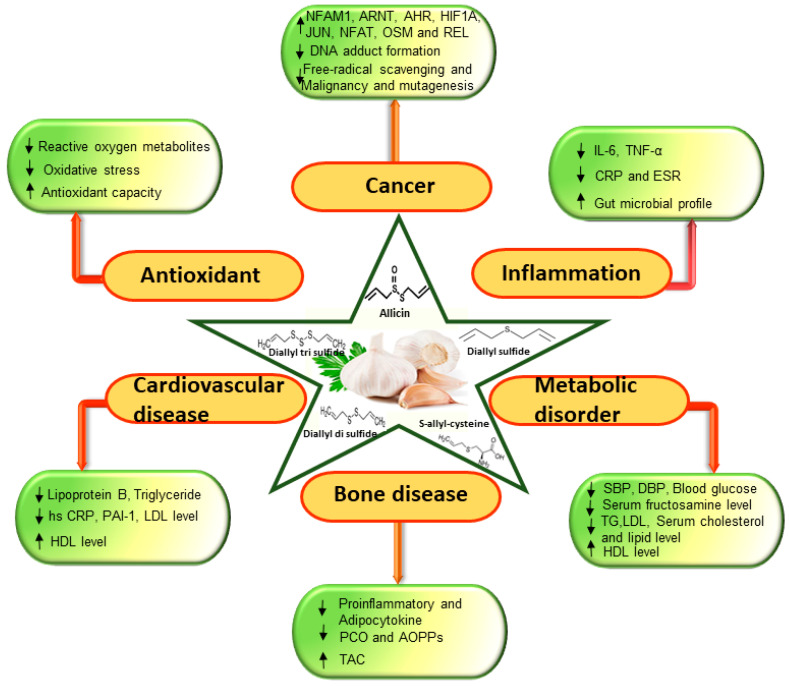
Schematic representation of garlic modulation of biomarkers in cancer, antioxidant activities, cardiovascular disease, bone, inflammation, and metabolic disorders in clinical trials. The symbol (↓) denotes reduced activity and (↑) denotes increased activity.

This work highlights garlic as a promising candidate for preventing and treating different health conditions. This review has summarized the anticancer, cardioprotective, antihyperglycemic, antimicrobial, antihypertensive, and others effect of the administration of garlic and its preparation through their antioxidant, anti-inflammatory and lipid-lowering activities (Figure 2). 

Garlic has been shown to modulate several biomarkers in different diseases in a multiple ways, however, to understand the exact mechanisms, it is necessary to perform large, long-term, fully blinded and well-controlled studies to obtain more precise and consistent findings. Additionally, further studies are needed to determine pharmacokinetic and pharmacodynamic limits in humans, such as pharmacologically active concentrations of garlic-derived sulfur compounds in garlic preparation that may be achieved via oral intake or through pharmacological interventions. In addition, the rapid metabolism and poor bioavailability of garlic are responsible for limiting its therapeutic use. Special attention needs to be focused to improve the bioavailability of garlic for the development of novel dosage. Finally, limited clinical evidence exists for the effects of garlic on neurodegenerative disorders, including Alzheimer’s disease, Parkinson’s disease, Huntington’s disease, and amyotrophic lateral sclerosis. This review could be helpful for future research priorities on garlic to be used in medicine, providing a wide range of health benefits.

## Figures and Tables

**Table 1:** Summary of clinical trial of garlic on antioxidant activities, anti-inflammatory properties, and lipid lowering effects.

Study Design	Patients	Intervention	Duration	Outcome	Mode of Action	Ref.
**Antioxidant activity**						
Randomized, double-blind, placebo-controlled trial	92 obese patients	400 mg of GE per day	3 months	-Increased total antioxidant status	-Modulation of hsCRP, LDL, HDL levels, triglycerides and PAI-1	[33]
Controlled trial	20 type 2 diabetes mellitus patients	3.6 g clove per day	30 days	-Enhanced antioxidant capacity	-Increased SOD, CAT and GPx concentration in circulating human erythrocytes	[34]
Double-blind randomized controlled clinical trial	42 menopausal women	2 garlic tablets equivalent 1200 μg Allcin per day	1 year	-Reduced oxidative stress	-Increase of TAC -Decreased MDA	[36]
Randomized controlled trial	46 untrained boys	250 mg garlic capsule per day	8 weeks	-Enhanced resistance and endurance training effect against oxidative stress	-Decreased MAD -Increased antioxidant capacity	[37]
Double blind, placebo-controlled crossover pilot study	26 patients with type 2 diabetes	1200 mg of AGE	4 weeks	-No significant effect on endothelial function and oxidative stress	-Not specified	[38]
Randomized, placebo-controlled, cross-over design	15 patients with CAD	2.4 g per day	2 weeks	-Improved impaired endothelial function -Reduced oxidative stress	-Increased FMD	[39]
Randomized clinical trial	20 patients with cancer	4.40 mg of allicin per g of garlic or 64-80 g per day	21–61 days	-Improved antioxidant status in cancer patients undergoing chemotherapy	-Decreased derivatives of reactive oxygen metabolites	[40]
Pilot study	20 healthy overweight adults	14.5 g spices per day	1 week	-Attenuated postprandial lipemia	-Inhibition of PL and PLA2	[41]
Randomized, double-blind, placebo-controlled trial	44 pregnant women	400 mg garlic and 1 mg allicin per day	9 weeks	-Reduced oxidative stress	-Increase of GSH -Decrease of hs-CRP	[42]
**Anti-inflammatory properties**						
Double blind randomized clinical trial	42 patients with peritoneal dialysis	400 mg of GE 2 times per day	8 weeks	-Anti-inflammatory effects in ESRD patients	-Decreased IL-6, CRP and ESR	[44]
Randomized, double-blind, placebo-controlled parallel-intervention study	120 healthy human	2.56 g AGE supplement per day	90 days	-Boost of immune system functions	-Not specified	[47]
Randomized, double-blind, placebo-controlled nutrition intervention	120 healthy subjects	2.56 g per day	90 days	-Enhanced immune cell activity -Reduced severity of colds and flu	-Not specified	[48]
Randomized controlled trial	60 healthy volunteers	1 g or 3 g of dehydrated garlic powder	6.0 and 24.0 h	-Immunostimulatory role in the urinary tract	-Improved urinary levels of IL-12	[16]
Randomized, double-blind, placebo-controlled clinical trial	51 healthy adults with obesity	3.6 g AGE per day	6 weeks	-Reduced obesity-induced inflammation	-Release of H_2_S from SAC via increasing its endogenous products.	[49]
Double-blind randomized placebo-controlled trial	49 patients with uncontrolled hypertension	1.2 g aged GE per day	12 weeks	-Improved inflammation and gut microbial profile	-Increased microbial richness and diversity -Improvement of immune system stimulating bacteria, *Lactobacillus,* and *Clostridia* species	[50]
Epidemiologic studies	90 overweight patients	2.1 g per day	12 weeks	-No significant effect on inflammatory biomarkers, endothelial function, or lipid profile	-Not specified	[51]
Double blind, placebo-controlled crossover pilot study	26 patients with type 2 diabetes	1200 mg AG per day	4 weeks	-No remarkable improvement in vascular inflammation	-Not specified	[38]
**Lipid lowering effects**						
Randomized control trial	160 type 2 diabetic patients	1.1 mL of olive oil and 500 mg of garlic powder	3 months	-Prevented dyslipidemia	-Decreased serum cholesterol and serum TG level	[53]
Double-blind, randomized placebo-controlled trial	60 patients with mild hypercholesterolemia	6 g ABG 2 times per day	12 weeks	-Decreased atherogenic markers -No significant effects on triglycerides, low-density lipoprotein cholesterol, total cholesterol, or free fatty acid levels -Shown cardio-protective effect	-Not specified	[55]
Double-blinded placebo-controlled study	60 patients with type 2 diabetes mellitus	Allicor 300 mg 2 times daily	4 weeks	-Maintained plasma lipid profile	-Increase of HDL -Reduction of TC, TG, and LDL	[56]
Randomized, single-blind, placebo controlled study	70 patients with dyslipidemia	Garlic tablet 300 mg 2 times daily	12 weeks	-Improvement of dyslipidemia	-Decrease of LDL and increased HDL levels	[57]
Randomized, double-blind, placebo-controlled trial	75 healthy human	10,8 mg alliin per day	12 weeks	-Lipid-lowering effects	-Decreased triacylglycerol concentration	[58]
Single-blind, placebo-controlled study	150 hyperlipidemic patients	400 mg garlic and 1 mg allicin in tablet 2 times daily	6 weeks	-Lipid-lowering effects	-Reduced cholesterol and LDL levels and increased HDL level	[59]

Abbreviations: ABG, aged black garlic; AGE, aged garlic extract; CAD, coronary artery disease; CAT, catalase; CRP, C-reactive protein; ESR, erythrocyte sedimentation rate; ESRD, end-stage renal disease; FMD, flow mediated endothelium-dependent dilation; GE, garlic extract; GPx, glutathione peroxidase; GSH, glutathione; IL-6, interleukin 6; HDL, high-density lipoprotein; LDL, low-density lipoprotein cholesterol; MAD, malondialdehyde; PAI-1, plasminogen activator inhibitor-1; PLA2, phospholipase A2; SAC, S-allyl cysteine; SOD, superoxide dismutase; TAC, total antioxidant capacity; TC, total cholesterol; TG, triglyceride.

**Table 2:** Summary of clinical trials of garlic on cancer, cardiovascular diseases, metabolic syndrome, and blood pressure.

Study Design	Patients	Intervention	Duration	Outcome	Mode of Action	Ref.
**Cancer**						
Personalized nutritional intervention study	20 patients	4.40 mg of allicin per g of garlic or 64-80 g per day	at least 3 weeks	-Improved antioxidant status	-Not specified	[40]
Randomized intervention trial	153 patients with breast cancer	39.0%–69.5% of garlic diet for 4 times per week.	6 months	-Increased adherence to a Mediterranean style	-Not specified	[63]
Randomized trial	3365 patients with gastric lesions	200 mg 2 caps or steam-distilled garlic oil 1 mg 2 time per day	7.3 years	-No significant effects on prevention of precancerous gastric lesions or on gastric cancer incidence	-Not specified	[64]
Double-blind intervention study	5033 patients with gastric cancer	200 mg synthetic allitridum per day and 100 µg selenium	1 month, each 3 years	-Protection from gastric cancer	-Reduction of tumor malignancy	[65]
Factorial placebo-controlled trial	3365 patients with gastric or esophageal cancer	400 mg of Kyolic aged GE 2 times per day	7.3 years	-No significant decrease of gastric cancer	-Not specified	[66]
Factorial, double-blind, placebo-controlled trial	4326 individuals with gastric lesion	AGE 400 mg, 2 times per day and steam-distilled garlic oil 2 mg, 2 times per day	3.5 and 7.5 years	-Reduction of burden of gastric cancer in high risk areas	-Not specified	[67]
Blinded randomized placebo controlled trial	3365 volunteers with H pylori positive participants and risk for gastric cancer	200 mg AGE and 1 mg steam distilled garlic oil 2 times daily	7.3 years	-Decreased risk of gastric cancer incidence and mortality	-Not specified	[68]
Randomized, double-blind, placebo-controlled factorial trial	3411 patients with gastric lesion	2 capsules 2 times per day	7.3 years	-Increased serum folate concentration	-Not specified	[69]
Comparison based study	57,560 men and women with colorectal cancer	One bulb per day	9 years	-Reduced risk of colorectal adenoma	-Not specified	[70]
Hospital-based matched case-control study	966 men and 700 women	<0.60 to >3.65 kg per year for garlic <0.60 to >3.0 kg per year for garlic stalks	2 years	-Reduced CRC risk in both men and women	-Not specified	[71]
Randomized double-blind trial	42 patients with liver cancer, 7 patients with pancreatic cancer and 1 patient with colon cancer	4 caps per day	6 months	-No improvement of quality of life	-Increased natural-killer cell activity	[73]
Population-based case-control study	5967 patients	Raw garlic 8.4 g per week or garlic components 33.4 g per week	7 years	-Chemopreventive effect	-Inhibition of mutagenesis by hindering the metabolism -Inhibition of DNA adduct formation, free-radical scavenging and effects on cell proliferation and tumor growth	[74]
A placebo-controlled double blind randomized study	95 patients	900 mg per day	3 weeks	-Protective effect in the lower-risk subgroup but no effects in the entire cohort	-Not specified	[75]
Randomized crossover feeding trial	17 volunteers	5 g raw, crushed garlic per day	For 10 days	-Activation of genes related to immunity, apoptosis, and xenobiotic metabolism	-Upregulation of NFAM1, ARNT, AHR, HIF1A, JUN, NFAT, OSM, and REL genes	[76]
**Cardiovascular diseases**						
Double-blinded placebo-controlled randomized study	51 patients with coronary heart disease	150 mg garlic tablet 2 times per day	12 months	-Decreased of cardiovascular risk by 1.5-fold in men and by 1.3-fold in women	-Reduced LDL cholesterol	[83]
Randomized trial	65 patients	250 mg AGE per day	12 months	-Decreased progression rate of adipose tissue volumes	-Reduction of EAT, PAT, PaAT, and SAT	[84]
Randomized, double-blinded trial	22 patients with risk for CVD	2400 mg AGE per day	1 year	-Increased microcirculation	-Not specified	[85]
A double-blind, placebo-controlled clinical trial	157 asymptomatic postmenopausal women	500 mg isoflavonoid containing garlic herbal preparation	12 months	-Prevention of atherosclerosis progression -Suppressed the formation of new atherosclerotic lesions approximately by 1.5-fold and decreased progression of existing ones	-Not specified	[86]
Randomized double-blind study	55 metabolic syndrome patients	2400 mg AGE per day	52 weeks	-Decreased low attenuation plaque in coronary arteries	-LAP percentage change	[87]
Randomized double-blind placebo-controlled nutritional intervention	92 obese patients	400 mg of GE per day	3 months	-Modified endothelial biomarkers associated with cardiovascular risk-Suppressed chronic inflammation	-Reduction of hsCRP, PAI-1, LDL cholesterol, and TAS	[33]
A randomized controlled trial	60 patients with mild hypercholesterolemia	6 g 2 times per day	12 weeks	-Decreased atherogenic markers -Exhibited cardioprotective effect	-Increased high-density lipoprotein cholesterol levels -Decreased levels of lipoprotein B	[55]
Randomized, placebo-controlled trial	10 patients with severe coronary artery disease	1200 mg 2 times per day	3 months	-Reduced progression rate of adipose tissue volumes	-Decrease of C-reactive protein levels -Improvement of brachial FMD values	[89]
A randomized clinical trial	62 healthy volunteers	1250 mg per day	1 month	-Garlic tablet did not have significant effect on PA -Shown mild adverse effect.	-Not specified	[91]
A double-blind placebo-controlled crossover study	14 healthy volunteers	9.9 g garlic	2 weeks	-Showed little or no effect in the reduction of platelet aggregation	-Not specified	[92]
Double-blind, randomized, placebo-controlled trial pilot study.	66 patients	AGE 5 mL 2 times per day	12 weeks	-Increased anticoagulation effect along with warfarin	-Not specified	[93]
**Metabolic syndrome**						
Randomized controlled trial	40 patients with metabolic syndrome	100 mg/kg body weight raw crushed garlic 2 times per day	4 weeks	-Decreased factors of metabolic syndrome	-Reduction of systolic and diastolic blood pressure and fasting blood glucose -Increased serum high-density lipoprotein cholesterol	[95]
Double-blinded placebo-controlled study	60 diabetic patients	300 mg Allicor 2 times per day	4 weeks	-Improved metabolic control	-lowering of fasting blood glucose, -Reduction of serum fructosamine and serum triglyceride levels	[56]
Controlled trial	20 diabetic patients	3.6 g clove per day	30 days	-Decreased blood glucose level	-Decreased lipid metabolism -Reduced serum cholesterol, TG and LDL -Improved HDL level	[34]
Controlled trial	Obese patients	Garlic pods 100 mg per day	5 months	-Increased hypoglycemic and hypolipidemic effect	-Decrease of blood glucose, cholesterol and triglycerides -Increase of HDL levels	[96]
Double-blind, crossover, randomized, placebo-controlled clinical trial	48 patients	1.2 g per day of AGE (Kyolic)	24 weeks	-Prevent cardiovascular complications in individuals with metabolic syndrome	-Increased the plasma levels of adiponectin	[97]
Placebo-controlled double-blind study	55 patients with metabolic syndrome	2400 mg AGE per day	52 weeks	-Decreased low attenuation plaque	-Not specified	[87]
**Blood pressure**						
A dose–response trial	79 patients with uncontrolled systolic hypertension	240/480/960 mg of aged garlic containing 0.6/1.2/2.4 mg of S-allylcysteine daily	12 weeks	-Antihypertensive effect	-Not specified	[100]
A double-blind crossover study	41 moderately hypercholesterolemic patients	7.2 g AGE per day	10 months	-Decreased systolic and diastolic blood pressure	-Decreased serum TC, LDL level	[101]
Randomized study	40 patients	100 mg/kg 2 times per day	4 weeks	-Reduced SBP and DBP	-Decreased of triglycerides -Increase of serum high-density lipoprotein cholesterol	[95]
Randomized, double-blind, placebo-controlled study	34 patients with prehypertension and 47 with mild hypertension	300 mg per day	12 weeks	-Decreased systolic BP and diastolic BP	-Not specified	[103]
Randomized study	100 patients	2 g per day	60 days	-Improved lipid parameters and reduced blood pressure	-Decrease of BMI, TC, and LDL -Increase of HDL	[104]
A double-blind, placebo controlled, randomized crossover study	28 patients with hypercholesterolemia	300 mg three times per day	28 days	-No significant effect of garlic ingestion on lipids and lipoproteins.	-Not specified	[105]

Abbreviations: AGE, aged garlic extract; AHR, aryl hydrocarbon receptor; ARNT, aryl hydrocarbon receptor nuclear translocator; BMI; body mass index; BP, blood pressure; CCTA, cardiac computed tomography angiography; CRC, colorectal cancer; CVD, cardiovascular disease; DBP, diastolic blood pressure; EAT, epicardial adipose tissue; FMD, flow mediated endothelium-dependent dilation; FN, febrile neutropenia; GE, garlic extract; HIF1A, hypoxia-inducible factor 1α; HDL, high-density lipoprotein; JUN, c-Jun; LAP, low-attenuation plaque; LDL, low-density lipoprotein cholesterol; NCP, noncalcified plaque; NFAM1, immunoreceptor tyrosine-based activation motif 1; NFAT, nuclear factor of activated T cells; OSM, oncostatin M; PaAT, periaortic adipose tissue; PAI-1, plasminogen activator inhibitor-1; PAT, pericardial adipose tissue; REL, V-rel avian reticuloendotheliosis viral oncogene homolog; SAT, subcutaneous adipose tissue; SBP, systolic blood pressure; TAS, total antioxidant status; TG, triglyceride.

**Table 3:** Summary of clinical trials of garlic on diabetes, bone, and skin diseases.

Study Design	Patients	Intervention	Duration	Outcome	Mode of Action	Ref.
**Diabetes**						
Single-blind placebo-controlled study	210 patients with type 2 diabetes mellitus	-Garlic tablets 300 mg per day -Garlic tablets 600 mg per day -Garlic tablets 900 mg per day -Garlic tablets 1200 mg per day -Garlic tablets 1500 mg per day -Metformin 500 mg 2 times per day	24 weeks	-Reduced fasting blood glucose -Reduction of HbA1C	-Not specified	[112]
Randomized controlled trial	50 patients with type 2 diabetes	300 mg of garlic contain sachet 2 times per day	12 weeks	-Lowered serum lipids	-Improved serum triglyceride, total cholesterol, and low-density lipoprotein levels	[113]
Parallel study	60 patients with type 2 diabetes	Garlic tablet 300 mg thrice daily and Metformin 500 mg 2 times per day	24 weeks	-Improved antihyperlipidemic activity	-Decreased total cholesterol, LDL and triglycerides	[114]
Double blind, placebo-controlled crossover pilot study	26 patients with type 2 diabetes	1200 mg AGE daily	4 weeks	-Did not significantly improve insulin resistance	-Not specified	[38]
Double-blind clinical trial	76 patients with diabetes	750 mg capsule contained nettle leaf 20% (*w/w*), berry leaf 10% (*w/w*), onion and garlic 20% (*w/w*), fenugreek seed 20% (*w/w*), walnut leaf 20% (*w/w*), and cinnamon bark 10% (*w/w*) 3 times daily	12 weeks	-Reduced fasting glucose blood sugar -Decreased HbA1c level.	-Not specified	[115]
**Bone diseases**						
Randomized, double-blind, placebo-controlled, parallel-design trial	80 postmenopausal overweight or obese women with knee OA	500 mg garlic tablet 2 times daily	12 weeks	-Reduced pain severity	-Reduction in the proinflammatory adipocytokine, resistin	[116]
Randomized double-blind, placebo-controlled, parallel design trial	76 postmenopausal overweight or obese women	1000 mg per day	12 weeks	-Improved OA symptoms	-Not specified	[117]
Double-blind randomized controlled clinical trial	42 menopausal women	2 garlic tablets equivalent of 2 g fresh garlic per day	1 year	-Reduced oxidative stress	-Decreased PCO plasma levels -Reduced AOPP level -Increased TAC	[118]
Double-blind randomized controlled clinical trial	44 postmenopausal osteoporotic women	2 garlic tablets per day	8 months	-Immunomodulatory effect	-Reduced TNF-α levels	[36]
**Skin diseases**						
Randomized control study	50 patients with warts	Lipid GE 2 times per day	4 weeks	-Successfully cure of recalcitrant multiple common warts	-Non-significant increase in TNF-α	[120]
Randomized double-blind clinical trial	40 patients with denture stomatitis	Garlic aqueous solution at 40 mg/mL 3 times per day	4 weeks	-Improved erythematous lesions	-Not specified	[121]
Prospective non-randomized pilot study	10 men and 15 women with venous ulcers	Ointment of GE	7 weeks	-Antiseptic, anti-inflammatory and epithelizing effects -Reduced venous ulcer	-Not specified	[122]

Abbreviations: AOPP, advanced oxidation protein product; GE, garlic extract; HbA1C, hemoglobin A1c; LDL, low-density lipoprotein cholesterol; OA, osteoarthritis; PCO, plasma protein carbonyl; TAC, total antioxidant capacity; TNF-α, tumor necrosis factor alpha.

**Table 4:** Summary of clinical trials of garlic on other conditions and diseases.

Study Design	Patients	Intervention	Duration	Outcome	Mode of Action	Ref.
**Antimicrobial efficacy**						
Randomized double-blind controlled clinical trial	45 children	Rinse mouth with 2 mL garlic formulation per day or garlic with lime mouth rinses	2 weeks	-Effective alternative of NaF mouth rinse	-Not specified	[133]
Randomized controlled trial	110 women with itching or a burning sensation in the vaginal area	1500 mg of Garcin tablets and fluconazole tablets 150 mg daily	7 days	-Improved vaginitis	-Not specified	[134]
Randomized placebo controlled double-blind trial	63 asymptomatic women with culture-positive for Candida species	350 garlic tablets 2 time per day	14 days	-Reported adverse effects	-Not specified	[135]
**Acute respiratory viral infections**						
Double-blind placebo-controlled randomized trial	796 children	First stage Allicor 600 mg per daySecond stage Allicor 300 mg per day	5 months	-Prevention of acute respiratory infections with no side effects	-Not specified	[136]
**Nosocomial infections**						
Clinical trial	94 patients	400 mg per day	6 days	-Effective against highly susceptible nosocomial infection patients	-Not specified	[137]
**Antileishmanial and immuno-modulatory activity**						
Randomized clinical study	70 cutaneous leishmaniasis patients	1 time daily	6 and 8 weeks	-Anti-leishmanial activity and lesion recovery	-Not specified	[139]
**Cystic fibrosis**						
Pilot randomized controlled trial	34 patients with cystic fibrosis	Garlic capsules (656 mg of garlic oil macerate and 10 mg cardamom oil) per day	8 weeks	-No significant effect on Pseudomonas aeruginosa quorum sensing -Minor side effects	-Not specified	[140]
**Hepatic diseases**						
Double-blind, randomized, placebo-controlled trial	75 adults with elevated GGT	1.5 g per day	12 weeks	-Improved hepatic dysfunction	-Increased levels of GGT and ALT -Improved fatigue scale scores	[141]
**Chronic hepatitis**						
Double-blind, placebo-controlled clinical trial	88 patients with histologically confirmed chronic hepatitis	3 to 6 capsules per day. Each capsule contains 25 mg DDB plus 50 mg GO	7 weeks	-DDB plus GO lowered serum aminotransferase activities	-Decreased ALT and AST levels	[143]
**Non-alcoholic fatty liver disease**						
Randomized, double-blind, placebo-controlled trial	110 patients NAFLD	Garlic tablet 400 mg per day	15 weeks	-Reduced body weight and fat mass	-Not specified	[144]
A population-based study	24,166 patients with NAFLD	1–7 g per week		-NAFLD onset	-Not specified	[145]

Abbreviations: ALT, alanine aminotransferase; AST, aspartate transaminase; DDB, dimethyl-4,4’-dimethoxy-5,6,5’,6’-dimethylene dioxybiphenyl-2,2’-dicarboxylate; GGT, gamma-glutamyl transpeptidase; GO, garlic oil; NaF, sodium fluoride; NAFLD, nonalcoholic fatty liver disease; QS, quorum sensing.
